# The impact of pulmonary tuberculosis on immunological and metabolic features of diabetic patients

**DOI:** 10.3389/fimmu.2022.973991

**Published:** 2022-08-23

**Authors:** Haijun Chen, Li Su, Jinhua Bao, Kun Zhang, Yuze Li, Enuo Mao

**Affiliations:** ^1^ Department of Computed Tomography, Heilongjiang Provincial Hospital, Harbin, China; ^2^ Neuroscience Research Institute, Peking University Center of Medical and Health Analysis, Peking University, Beijing, China; ^3^ College of Sports and Human Sciences, Harbin Sport University, Harbin, China; ^4^ Department of Clinical Nutrition, Heilongjiang Provincial Hospital, Harbin, China; ^5^ Department of the Fourth Internal Medicine, The Fourth Hospital of Heilongjiang Province, Harbin, China; ^6^ Department of Discipline Inspection and Supervision, Heilongjiang Provincial Hospital, Harbin, China

**Keywords:** type-2 diabetes mellitus, pulmonary tuberculosis, cytokines, monocytes, interleukin 10

## Abstract

Impaired immune responses have been observed in patients with type-2 diabetes mellitus (T2DM), which increases susceptibility to tuberculosis infection. However, the effect of the tuberculosis infection on the immunological and metabolic features of T2DM is largely unknown. To investigate this question, age- and sex-matched patients with pulmonary tuberculosis (PTB), T2DM, or T2DM combined with PTB were recruited from the Infectious Disease Hospital of Heilongjiang Province between January and September 2020. Healthy subjects were used as controls. Cytokines and chemokines in fasting serum samples were determined using the Quantibody Inflammation Array. Compared with T2DM alone, patients with T2DM combined with PTB have higher fasting blood glucose levels and monocyte counts in circulation. Among the four groups, circulating IL-10 levels peaked in patients with T2DM and PTB (p<0.05). Univariate linear analysis showed that serum IL-10 levels were positively associated with myeloid cells but negatively correlated with lymphocyte counts in these patients (p<0.05). Serum IL-6 levels were 1.6-fold higher in patients with T2DM plus PTB than in those with T2DM alone. In conclusion, PTB infection in patients with T2DM had distinct inflammatory profiles and sustained hyperglycaemia compared with PTB or T2DM alone. IL-10 levels and elevated monocyte counts could be hallmarks of patients with T2DM infected with PTB.

## Introduction

The prevalence of type-2 diabetes (T2DM) has increased annually and has become a global burden. According to the latest diabetes epidemiologic report in 2020, there were a total of 130 million patients with diabetic, and the prevalence rate of adult diabetes was up to 12.8% in China (1). Similarly, there are approximately 10 million patients with pulmonary tuberculosis (PTB) worldwide, 8.4% of whom are residents of China (2). Notably, the risk of PTB infection in diabetic patients is 1-3 times higher than that in non-diabetic subjects (3). Clinical data have revealed that patients with diabetes and PTB are characterized by sustained hyperglycaemia and prolonged pulmonary infection compared with patients with PTB only, all of which adversely affect the effect of drug treatment and lead to a significant increase in recurrence rate and mortality (4). Therefore, it is essential to understand the mechanisms by which diabetes drives PTB progression for therapeutic purposes.

Physically, lung-resident macrophages serve as innate immune cells that protect against infection. The clearance in the lungs is mainly mediated through phagocytosis and inflammatory cytokines. Pathogen-associated molecular patterns (PAMPs) on PTB enable the recognition of foreign pathogens by receptors expressed on resident macrophages. Nucleotide oligomerization domain-like receptors (NLRs) are cytosolic pattern recognition receptors that recognize PTB PAMPs following phagocytosis, leading to the activation of inflammasome formation. In parallel, PTB expresses Toll-like receptor (TLR) ligands. Among all TLR ligands, TLR-2, TLR-4, and TLR-9 are known to be involved in the recognition. Downstream of the TLR signaling pathways, the innate immune response is further propagated. For instance, interleukin (IL) is one of the main cytokines which plays a key role in anti-tuberculosis immunity regulation (5, 6). Accumulating evidence illustrates the central role of resident macrophages in PTB clearance. However, PTB can modify macrophage maturation and reduce its antibacterial capacity. In this study, we explored the deleterious effects of PTB infection on glucose and lipid metabolism, as well as inflammation features in patients with T2DM.

To investigate this question, patients with T2DM alone, PTB alone, and PTB co-morbidity with T2DM were enrolled in the study. Healthy individuals were used as controls. The fasted plasma samples were subjected to panels of cytokine/chemokine measurements using the Quantibody Human Inflammation Array. The relationship between the cytokines of interest and the features of the diseases was investigated.

## Materials and methods

### Study patients

Patients with T2DM (n=12), PTB (n=14), or PTB combined with T2DM (n=12) admitted to the Infectious Disease Hospital of Heilongjiang Province between January and September 2020 were recruited for this study. Twelve healthy participants were used as controls. All study subjects were age- and sex-matched. Basic information was collected, including medical records, sex, age, body weight, and height. Body mass index (BMI) was calculated as the weight in kilograms divided by the square of height in meters. Hypertension was defined as a systolic blood pressure of at least 140 mmHg, diastolic blood pressure of at least 90 mmHg, or use of antihypertensive drugs.

The study was reviewed and approved by the institutional review board of Heilongjiang Provincial Hospital. All participants provided written informed consent before enrolment in the study.

### Inclusion and exclusion criteria

The inclusion criteria were as follows. They were from the Han population. Patients with T2DM were diagnosed with a fasting blood glucose level of at least 7.0 mmol/L or blood glucose levels exceeded 11.0 mmol/L or more after 2 h of oral administration of 75-g glucose. PTB was diagnosed when any of the following three conditions were met on sputum samples prepared by direct smear: (1) two sputum samples were acid-fast bacilli positive under microscopic examination; (2) one sputum sample was acid-fast bacilli positive on microscopic examination, and signs of active pulmonary tuberculosis were found on pulmonary imaging examination; (3) one sputum sample was acid-fast bacilli positive on microscopic examination, and another sputum sample was Mycobacterium tuberculosis positive after cultivation (7). The healthy control group had no history of diabetes or tuberculosis, and routine blood and urine examination, biochemistry, and other examinations were normal.

Patients infected with HIV, hepatitis virus, or syphilis or those with tumors were excluded from the study.

### Biochemical measurements

White blood cell and differential white blood cell counts were obtained. Lipid data comprising serum total cholesterol, triglyceride, high-density lipoprotein cholesterol, and low-density lipoprotein cholesterol levels were measured in the central laboratory of the hospital. Venous blood samples were analyzed for plasma glucose, creatinine, glutamyl transpeptidase, uric acid, total bilirubin, and bile acids. The glomerular filtration rate (eGFR) was derived from serum creatinine levels using the Chronic Kidney Disease Epidemiology Collaboration (CKD-EPI) equation (8). Low-density lipoprotein (LDL) cholesterol was computed from serum total and HDL-cholesterol, and serum triglycerides using the Friedewald equation (9).

### Serum sample collection and storage

Fasting peripheral blood samples were collected from all patients. Serum was collected after centrifugation at 3000 rpm for 10 min, and the supernatant was stored at -80°C.

### ELISA assay

Equal Fried Ewald amounts of plasma samples were subjected to cytokine/chemokine measurements according to the manufacturer’s instructions (Quantibody Human Inflammation Array, RayBiotech Inc, Guangzhou, China). Chip drying, gradient dilution of the cytokine standard, serum sample dilution, chip sample addition, cleaning, detection of antibody mixture incubation, and other steps were performed. The cytokines measured were GM-CSF, IFN-γ, IL-1β, IL-2, IL-4, IL-5, IL-6, IL-10, IL-12p70, IL-13, IL-17, IL-17F, IL-21, IL-22, IL-23, IL-28A, MIP-3a, TGF-β1, TNF-α, and TNF-β.

### Statistical analysis

R software was used for statistical analysis. The measurement data were normally distributed, and were expressed as “mean ± standard deviation”. A variance analysis was performed for inter-group comparisons. Least significant difference (LSD) test was used for further pairwise comparisons. The χ2 test was used to compare the sex distribution in the basic information of the study population. P < 0.05 was considered statistically significant.

## Results

### General characteristics of the study objects

**Figure 1:**
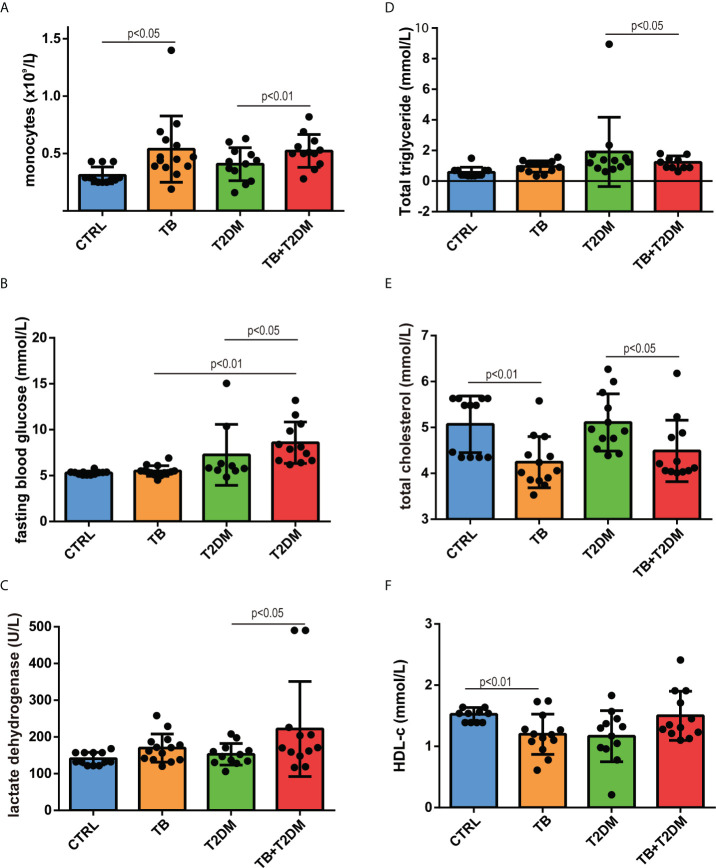
General features of the study subjects. **(A)** blood monocyte count; **(B)** fasting blood glucose; **(C)** serum lactate dehydrogenase; **(D)** serum triglyceride levels; **(E)** serum cholesterol levels; **(F)** HDL-cholesterol levels.

The general characteristics of the patients in this study are presented in **Table 1**. Compared with healthy controls, patients with PTB had higher blood monocyte counts, platelet counts, and serum triglyceride levels but lower total cholesterol and HDL-c levels (**Figure 1A**). A similar pattern was observed for T2DM alone and in combination with PTB. In addition, fasting blood glucose levels were 1.3-fold higher in T2DM patients with PTB than T2DM alone (**Figure 1B**). Serum levels of lactate dehydrogenase were higher in the patients with T2DM with PTB than those of the other groups, indicating more severe damage in the former group (**Table 1**; **Figure 1C**). With regard to the lipid profile, serum triglyceride levels were 1.4-fold higher in PTB patients than in healthy participants, but were comparable in patients with T2DM with or without PTB (**Figure 1D**). Moreover, total cholesterol and HDL-c levels were both 1.3-fold higher in patients with PTB than in healthy controls (**Figures 1E, F**). Likewise, total cholesterol levels were 1.2- and 1.1-fold higher in patients with T2DM with PTB than those in patients with T2DM alone. These data indicate that PTB infection affects lipid and glucose metabolism in patients with T2DM.

**Table 1:** General characteristics of the study subject.

	Healthy controls	PTB	P value	T2DM	PTB with T2DM	P value
**Number**	12	14		12	12	
**Male**	8	9	1.00	8	8	1.00
**Smoking**	0	5	0.20	5	0	0.04
**Hypertension**	2	/	NA	7	4	0.41
**Cardiovascular disease**	0	/	NA	3	2	1.00
**Mean (SD)**						
** Age (years old)**	59.90 (8.25)	58.15 (12.00)	0.094	59.91 (10.50)	60.15 (12.25)	0.95
** Body build index (kg/m^2^)**	22.89 (1.78)	20.50 (3.70)	<0.05	24.61 (3.02)^**^	21.91 (1.50)^*##^	<0.01
** Body Weight (kg)**	/	/	/	72.36 (11.26)	67.18 (13.56)	0.323
** Systolic pressure (mmHg)**	/	/		144.27 (21)	132.15 (16.25)	0.12
** Diastolic blood pressure (mmHg)**	/	/		84.94 (8)	84.24 (15.75)	0.45
** White blood cell count (x10^9^/L)**	5.13 (0.93)	6.36 (1.18)	0.014	5.98 (1.71)	6.21 (4.44)	0.66
** Neutrophils (x10^9^/L)**	3.00 (0.66)	4.00 (1.44)	0.032	3.39 (1.03)	3.86 (3.25)	0.71
** Monocytes (x10^9^/L)**	0.30 (0.06)	0.49 (0.22)^*^	0.002	0.38 (0.17)	0.50 (0.10)^**^	0.073
** Lymphocytes (x10^9^/L)**	1.75 (0.26)	1.56 (0.5)	0.353	1.95 (0.58)	1.43 (1.70)	0.25
** Einosinophils (x109/L)**	0.06 (0.02)	0.15 (0.09)	0.007	0.15 (0.07)	0.74 (0.11)	0.20
** Basophils (x10^9^/L)**	0 (0)	0.04 (0.04)	5.64x10^-6^	0.03 (0.01)	0.14 (0.03)	0.008
** Red blood cell count(x10^12^/L)**	4.47 (0.19)	4.25 (0.69)	0.216	4.51 (0.79)	4.37 (0.86)	0.73
** Blood platelet count (x10^9^/L)**	270.33 (28.4)	304.41 (82.8)^##^	0.643	219.76 (64.4)^**^	239.77 (87.8)^**##※※^	<0.0001
**Biochemistry data**						
** Serum creatinine(μmol/L)**	58.46 (6.38)	49.70 (15)	0.063	62.38 (22.16)	63.41 (12.75)	0.82
** EGFR (mL/min/1.73 m2)**	116.52 (12.5)	114.03 (15.84)	>0.05	97.98 (13.40)^*^	100.95 (23.41)^*^	>0.05
** Total cholesterol (mmol/L)**	5.03 (1.28)	4.16 (0.55)^**##^	0.004	5.07 (0.80)	4.45 (0.75)^*#^	0.013
** LDL-c (mmol/L)**	2.85 (0.93)	2.6 (0.62)	0.071	3.02 (0.66)	2.70 (1.12)	0.27
** HDL-C cholesterol (mmol/L)**	1.52 (0.25)	1.15 (0.19)^**^	0.006	1.05 (0.42)^*^	1.46 (0.54)^※^	0.11
** Serum triglyceride (mmol/L)**	0.51 (0.31)	0.80 (0.53)^*^	0.025	1.40 (0.67)	1.16 (0.64)^**^	0.75
** Fasting glucose (mmol/L)**	5.28 (0.26)	5.47 (0.2)	0.197	6.38 (0.64)	8.33 (3.41)^**※※^	0.012
** Total bile acid(umol/L)**	2.23 (1.88)	3.89 (2.9)	0.015	3.90 (3.55)	3.82 (2.47)	0.73
** Blood uric acid (umol/L)**	258.23 (11.9)	340.15 (223.8)	0.020	304.52 (89.2)^*^	346.32 (173.8)^**※^	0.27
** Total bilirubin (umol/L)**	7.24 (1.68)	8.60 (4.75)^##^	0.642	14.67 (6.08)^**^	8.18 (4.48)^##^	0.004
** Haemoglobin (g/L)**	120.00 (4.73)	117.00 (24.26)	0.717	141.00 (16.50)	135.40 (19.24)	0.544
** Total protein (g/L)**	75.39 (3.30)	70.29 (5.85)	0.053	69.19 (6.18)	66.08 (7.50)	0.285
** CO_2_ (mmol/L)**	25.75 (0.28)	25.41 (1.47)	0.003	25.35 (2.00)	27.00 (2.22)	0.060
** Alanine aminotransferase (IU/L)**	9.98 (1.51)	11.07 (11.04)	0.155	21.04 (15.03)	23.42 (23.43)	0.908
** Aspartate aminotransferase (IU/L)**	14.47 (2.15)	16.07 (12.06)	0.736	18.21 (10.88)	13.21 (7.76)	0.214
** Alkaline phosphatase (IU/L)**	47.39 (14.28)	72.29 (23.60)	0.014	72.28 (29.76)	82.67 (16.12)	0.112
** Adenosine deaminase (IU/L)**	9.43 (2.08)	14.43 (7.98)	0.022	14.35 (6.70)	23.84 (11.71)	0.061
** Glutamyl transpeptidase (IU/L)**	13.10 (4.32)	19.83 (14.5)	0.215	31.82 (15.10)	47.06 (48.75)	0.065
** Lactate dehydrogenase (U/L)**	140.16 (27.5)	166.02 (45.8)	0.030	150.37 (31.2)	197.48 (54.8)	0.11

eGFR refers to estimated glomerular filtration rate obtained from serum creatinine through chronic kidney disease epidemiological collaboration (CKD-EPI) equation; LDL-c, low density lipoprotein cholesterol; HDL-c, high density lipoprotein cholesterol. P value is used for tuberculosis, type 2 diabetes as well as the difference between tuberculosis and type-2 diabetes.※represents statistic difference compared with PTB group (P < 0.05);※※represents significant statistic difference compared with PTB group (P < 0.01).

### Ratio of myeloid cells versus lymphocytes peaked in T2DM patients with PTB

**Figure 2:**
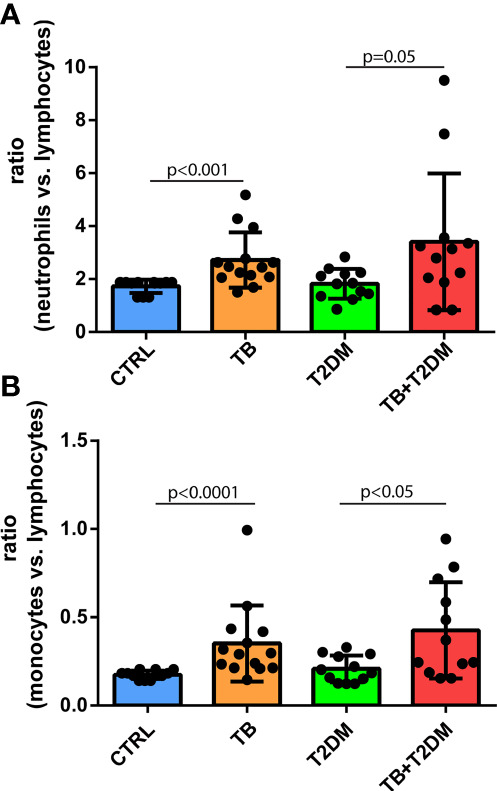
**(A)** The ratio of neutrophils to lymphocyte count in the study subjects; **(B)** The ratio of monocyte number to lymphocyte count in the study subjects.

A body of evidence has shown an increased ratio of myeloid cells to lymphocyte counts in patients with T2DM compared to non-diabetic participants (10). We performed a head-to-head comparison of the ratios among all groups. When considering the ratio of neutrophils to lymphocyte count in healthy participants as the reference, the ratio was elevated 1.57-, 1.05-, and 1.97-fold in PTB alone, T2DM alone, or T2DM combined with PTB, respectively (**Figure 2A**). In parallel, the ratio of monocyte number to lymphocyte count was 2.0-, 1.2-, and 2.4-fold higher in PTB alone, T2DM alone, or T2DM combined with PTB, respectively, compared with the control group (**Figure 2B**).

### Comparison of cytokine levels in the study groups

**Figure 3:**
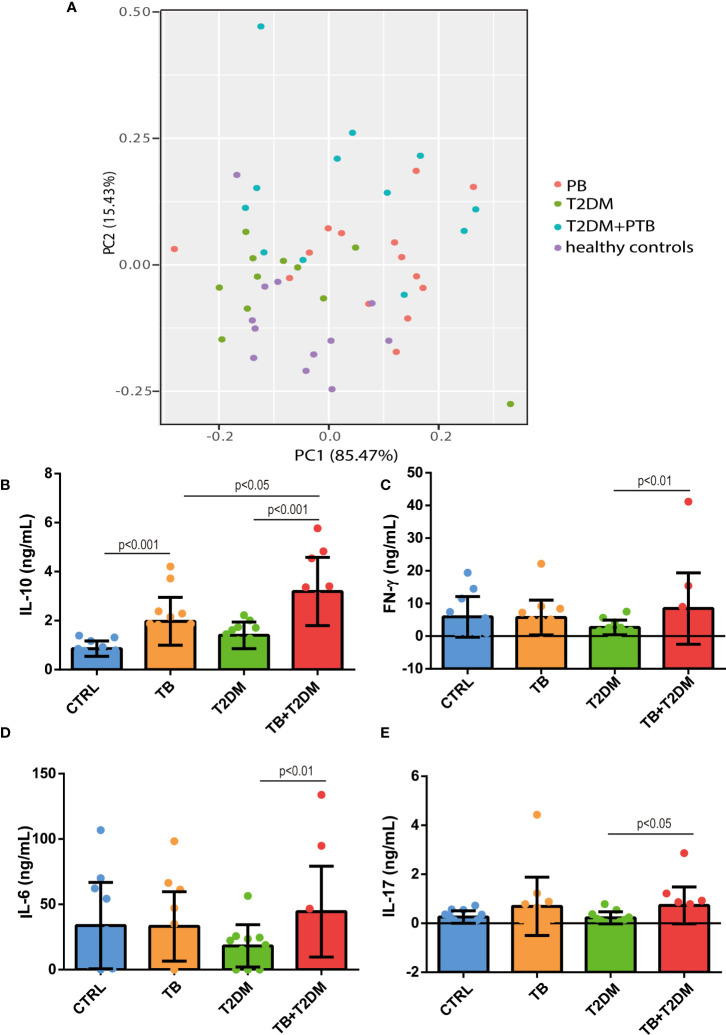
Serum levels of IL-10, IL-6, IL-17 and IFN-gamma among all groups. **(A)** PCA plot. **(B–E)** serum levels of IL-10, IFN-γ, IL-6 and IL-17 in the study subjects. CTRL, healthy controls; PTB, pulmonary tuberculosis; T2DM, type 2 diabetes mellitus; PTB+T2DM, pulmonary tuberculosis combined with type 2 diabetes mellitus.

Twenty inflammatory cytokines were identified. The PCA plot illustrates the distribution of each patient (**Figure 3A**). Serum levels of these cytokines are shown in **Table 2**. The relationships among all cytokines measured are shown in **Table 3**. Serum levels of IL-10 were 2.0-fold higher in the PTB group than in healthy controls. However, IL-10 levels were 1.9- and 2.0-fold higher in patients with T2DM combined with PTB than in PTB or T2DM alone, respectively (p<0.05) (**Figure 3B**). Similarly, an increase in the levels of IL-6, IL-17, and IFN-γ was observed between PTB and healthy controls, although the difference was not significant (p≥0.26). Nevertheless, they were all significantly higher in patients with T2DM with PTB than in T2DM patients alone (p<0.05) (**Figure 3C–E**).

**Table 2:** Immunity and inflammation characteristics of study objects in four groups .

Cytokines (ng/mL)	Healthy controls	Pulmonary tuberculosis (PTB)	P value	Type-2 diabetes mellitus (T2DM)	PTB plus T2DM	P value
**GM-CSF**	14.86 (8.22)	19.30 (14.73)	0.230	56.79 (9.34)	16.92 (6.99)	0.266
**IFN-γ**	5.91 (7.50)	5.71 (3.91)	0.74	10.51 (3.09)	8.45 (3.44)	0.039
**IL-1β**	10.96 (15.17)	18.26 (11.12)	0.53	58.49 (10.10)	21.26 (12.46)	0.93
**IL-2**	8.21 (6.61)	6.63 (1.88)	0.60	12.39 (2.78)	8.019 (3.32)	0.16
**IL-4**	1.83 (1.81)	1.08 (1.10)	0.90	11.11 (1.71)	1.06 (1.14)	0.76
**IL-5**	2.22 (1.41)	2.15 (1.37)	0.46	2.95 (1.80)	2.79 (2.81)	0.44
**IL-6**	33.78 (45.47)	33.15 (27.77)	0.92	43.94 (14.66)	36.52 (17.87)^##^	0.040
**IL-10**	0.91 (0.57)	1.78 (0.85)^**^	0.002	1.61(0.64)	3.32 (2.31)^**##^	0.003
**IL-12p70**	1.14 (1.08)	0.80 (1.08)	0.78	6.06 (2.10)	1.91 (0.62)	0.98
**IL-13**	2.12 (1.51)	1.49 (3.40)	0.74	1.55 (3.66)	1.93 (1.72)	0.59
**IL-17**	0.26 (0.41)	1.80 (0.82)	0.26	0.36 (0.35)	0.73 (0.54)	0.046
**IL-17F**	10.59 (9.67)	92.79 (98.46)	0.0054	175.46 (10.76)	111.45 (94.11)	0.16
**IL-21**	728.5 (441.0)	590.5 (582.8)	0.40	413.4 (416.8)	456.0 (416.8)	0.55
**IL-22**	40.26 (26.95)	25.99 (31.40)	0.274	55.68 (36.10)	32.87 (24.38)	0.590
**IL-23**	16.84 (9.77)	8.37 (10.03)	0.117	26.93 (3.12)	23.35 (9.15)	0.068
**IL-28A**	4.86 (2.58)	4.92 (1.34)	0.051	18.46 (1.16)	2.37 (1.96)	0.303
**MIP-3a**	1.15 (1.13)	1.59 (1.43)	0.297	3.854 (2.318)	2.77 (4.00)	0.755
**TGF-β1**	369.3 (380.7)	382.0 (825.6)	0.349	8.63 (28.64)	84.02 (101.7)	0.015
**TNF-α**	26.77 (37.25)	14.79 (23.87)	0.438	64.27 (10.35)	67.18 (46.76)	0.192
**TNF-β**	1.081 (0.83)	0.78 (0.71)	0.625	1.68 (1.04)	1.18 (0.84)	0.977

Data were expressed as mean (SD). * represents statistic difference compared with JK group (P < 0.05); ** represents significant statistic difference compared with JK group (P < 0.01); # represents statistic difference compared with T2DM group (P < 0.05); ##represents significant statistic difference compared with T2DM group (P < 0.01); ※※ represents statistic difference compared with PTB group (P < 0.05);※※※ represents significant statistic difference compared with PTB group (P < 0.01).

**Table 3:** Correlation matrix.

	IL-1β	IL-2	IL-4	IL-5	IL-6	IL-10	IL-12p70	IL-13	IL-17	IL-17F	IL-21	IL-22	IL-23	IL-28A	GM-CSF	IFN-γ	MIP-3α	TGF-β1	TNF-α	TNF-β
**IL-1β**	1	0.70‡	0.54‡	0.20	0.32*	0.12	0.64‡	0.72‡	0.24	0.15	0.55‡	0.83‡	0.38†	-0.07	0.04	0.52†	0.23	-0.02	0.24†	0.43†
**IL-2**	0.70‡	1	0.52†	0.42†	0.55‡	0.17	0.51†	0.59‡	0.45†	0.23	0.47†	0.65†	0.66†	0.18	0.40†	0.82†	0.31*	0.28*	0.65†	0.60†
**IL-4**	0.54‡	0.52‡	1	0.25	0.60‡	0.11	0.80‡	0.58‡	0.40#	0.13	0.38#	0.65‡	0.27	-0.01	0.19	0.45†	0.17	0.14	0.52‡	0.46†
**IL-5**	0.20	0.42†	0.25	1	0.45†	0.39†	0.31*	0.44†	0.40†	0.07	0.24	0.26	0.25	0.17	0.70‡	0.50‡	0.12	0.18	0.37#	0.32*
**IL-6**	0.32*	0.55‡	0.60‡	0.45†	1	0.37#	0.47†	0.33*	0.54‡	0.16	0.25	0.38#	0.41#	0.20	0.46†	0.68‡	0.30*	0.43†	0.60‡	0.45†
**IL-10**	0.12‡	0.17	0.11	0.39†	0.37#	1	0.26	0.19	0.36*	0.10	0.04	-0.11	-0.02	0.01	0.38#	0.28	0.39#	-0.04	0.25	0.22
**IL-12p70**	0.64‡	0.51‡	0.80‡	0.31*	0.47†	0.26	1	0.73‡	0.48‡	0.12	0.45†	0.72‡	0.29*	-0.04	0.15	0.45†	0.15	0.06	0.53‡	0.54‡
**IL-13**	0.72‡	0.59‡	0.58‡	0.44†	0.33*	0.19	0.73‡	1	0.35*	0.23	0.59‡	0.70‡	0.46†	0.13	0.22	0.52‡	0.11	0.12	0.45†	0.42†
**IL-17**	0.24	0.45†	0.40#	0.40†	0.54‡	0.36*	0.48‡	0.35*	1	0.27	0.18	0.32*	0.70‡	0.05	0.58‡	0.45†	0.17	0.20	0.52‡	0.42†
**IL-17F**	0.15	0.23	0.13	0.07	0.16	0.10	0.12	0.23	0.27	1	0.10	0.13	0.30*	-0.12	0.22	0.18	0.15	0.03	0.09	0.27
**IL-21**	0.55‡	0.47†	0.38#	0.24	0.25	0.04	0.45†	0.59‡	0.18	0.10	1	0.48‡	0.45†	0.26	0.23	0.41†	0.15	0.49‡	0.36*	0.33*
**IL-22**	0.83‡	0.65‡	0.65‡	0.26	0.38#	-0.11	0.72‡	0.70‡	0.32*	0.13	0.48‡	1	0.45†	-0.01	0.05	0.52‡	0.26	0.02	0.56‡	0.59‡
**IL-23**	0.38#	0.66‡	0.27	0.25	0.41#	-0.02	0.29*	0.46†	0.35*	0.30*	0.45†	0.45†	1	0.33*	0.26	0.52‡	0.25	0.50‡	0.53‡	0.42†
**IL-28A**	-0.07	0.18	-0.01	0.17	0.20	0.01	-0.04	0.13	0.05	-0.12	0.26	-0.01	0.33*	1	0.20	0.14	0.10	0.46†	0.23	0.08
**GM-CSF**	0.04	0.40#	0.19	0.70‡	0.46†	0.38#	0.15	0.22	0.58‡	0.22	0.23	0.05	0.26	0.20	1	0.44†	0.08	0.35*	0.30*	0.30*
**IFN-g**	0.52‡	0.82‡	0.45†	0.50‡	0.68‡	0.28	0.45†	0.52‡	0.45†	0.18	0.41†	0.52‡	0.52‡	0.14	0.44†	1	0.32*	0.27	0.60‡	0.58‡
**MIP-3a**	0.23	0.31*	0.17	0.12	0.30*	0.39#	0.15	0.11	0.17	0.15	0.15	0.26	0.25	0.10	0.08	0.32*	1	-0.03	0.39#	0.37#
**TGF-β1**	-0.02	0.28*	0.14	0.18	0.43†	-0.04	0.06	0.12	0.20	0.03	0.49‡	0.02	0.50‡	0.46†	0.35*	0.27	-0.03	1	0.12	0.15
**TNF-α**	0.50‡	0.65‡	0.52‡	0.37#	0.60‡	0.25	0.53‡	0.45†	0.52‡	0.09	0.36*	0.56‡	0.53‡	0.23	0.30*	0.60‡	0.39#	0.12	1	0.66‡
**TNF-β**	0.44†	0.60‡	0.46†	0.32*	0.45†	0.22	0.54‡	0.42#	0.42†	0.27	0.33*	0.59‡	0.42†	0.08	0.30*	0.58‡	0.37#	0.15	0.66‡	1

Significance: *p < 0.05, #p < 0.01; †p < 0.001; ‡p < 0.0001.

### Relationship between cytokines and blood monocyte count

We detected differential expression of serum IL-10 among all the groups. Finally, we examined the relationship between IL-10 levels, white blood cell counts, and biochemical parameters. Univariate analysis showed that serum IL-10 was positively associated with the proportions of monocytes, neutrophils, eosinophils, basophils, fasting blood glucose, and triglycerides, but negatively correlated to lymphocyte proportion, platelet count, and total cholesterol (p<0.05) (**Table 4**). Except for serum IL-17 levels, which were positively correlated with total triglyceride levels, no significant association was observed between IL-17 and the parameters mentioned above (TG). Moreover, neither IL-6 nor IFN-γ levels were associated with these parameters (p≥0.16). Considering that all subjects were age- and sex-matched among groups, multivariate-adjusted analysis was not further processed.

**Table 4:** Univariate correlation analysis in related to IL-10 levels.

Parameters	r	p value
N	46	
White blood cells (x10^9^/L)	0.26	0.09
Lymphocytes (%)	-0.42	0.004
Monocytes (%)	0.27	0.069
Neutrophils (%)	0.30	0.047
Eosinophils (%)	0.39	0.007
Basophils (%)	0.40	0.006
Platelet (x10^12^/L)	-0.55	<0.0001
Fasting blood glucose (mmol/L)	0.43	0.004
Total cholesterol (mmol/L)	-0.37	0.01
Total triglyceride (mmol/L)	0.37	0.01

## Discussion

The main findings of the study are summarized as follows: (1) compared to healthy controls, patients with PTB had low BMI and skewed myelopoiesis, as evidenced by increased myeloid cell number but reduced lymphocyte count. In addition, serum triglyceride levels were higher, but both total cholesterol and HDL-c levels were lower in patients with PTB than in healthy controls. (2) To some extent, when combined with T2DM, patients with PTB experienced increased myelopoiesis and reduced total cholesterol and HDL-c levels compared with T2DM alone. More strikingly, T2DM comorbid with PTB suffered from greater hyperglycaemia and liver dysfunction than T2DM alone, as shown in **Table 1**; and (3) Serum IL-10 levels peaked in patients with T2DM combined with PTB among all groups. Taken together, these data indicate that the presence of PTB attenuates T cell-mediated immune responses but reinforces the progression of skewed myelopoiesis and metabolic disorders in patients with T2DM.

Patients with T2DM manifested with obesity, lipid disorders, and low-grade chronic inflammation. Accumulating evidence suggests that immune cells are the main driver of metabolic disorders and inflammation, leading to adverse vascular outcomes. Data from patients and mice with diabetes have consistently demonstrated an increased ratio of myeloid cells to lymphocytes, which further increases with disease progression (11, 12). In addition, intracellular levels of GM-CSF and IL-6 were higher in the bone marrow cells of db/db mice than in age-matched wild-type controls (12), indicating the pathological basis of skewed myelopoiesis. In line with this, under sustained inflammatory conditions, infiltrating macrophages produce cytokines, such as IL-1β which promotes β cell dysfunction (13). In contrast, inhibition of macrophage recruitment by clodronate liposomes improves β-cell function and glucose tolerance in db/db mice (13). Advanced and active plaques with enriched macrophages, T lymphocytes, and larger necrotic cores have been observed in the coronary arteries of patients with diabetes than in non-diabetic individuals, which poses vulnerability to plaque rupture and a high risk of adverse cardiovascular outcomes in these patients (14).

Upon PTB infection, they replicate in alveolar macrophages and then spread to macrophages, myeloid dendritic cells, and neutrophils that are recruited from the periphery to further promote phagocytosis. The activation of complementary component C3 enhances the adherence and uptake of M. tuberculosis to facilitate mononuclear phagocytes and B-lymphocyte activation for antibody production. Activated by antigen-presenting cells, antigen-specific T-cell clones are expanding and home-to-lung for pathogen clearance. Taken together, innate and adaptive immune systems are well-coordinated to fight PTB (15–17). Nevertheless, the immune system is dysfunctional in patients with diabetes, making them highly susceptible to PTB infection. For instance, reduced production of IFN-γ, IL-1β, IL-12, and IL-18 was detected in alveolar macrophages from db/db mice that were infected with PTB compared to non-diabetic cells (15). Therefore, it is of great importance to explore whether and how PTB affects patients with diabetes for therapeutic purposes.

Consistent with other reports (18, 19), fasting blood glucose levels in patients with T2DM combined with PTB were higher than those of other groups in our study, indicating that more effort is required to achieve glucose homeostasis in these patients. Monocyte numbers peaked in patients with T2DM with PTB infection compared to the other groups. The monocyte-to-lymphocyte ratio seems to be a stronger hallmark in patients with T2DM with PTB than the neutrophil-to-lymphocyte ratio (**Figure 2**). Peripheral blood monocytes aggregate in the alveoli, where they develop into macrophages (20–22) after infection with M. tuberculosis. As a result, interleukins are produced, which play a role in the protective immune response of the body (22–25).

IL-10 is an important inflammatory immunosuppressive cytokine during tuberculosis immunity that can inhibit the formation of phagosomes in macrophages and weaken the antigen-presenting effect of macrophages, thereby reducing the clearance effect of the immune system against M. tuberculosis (26, 27). In our study, serum IL-10 levels were significantly increased (28) in patients with T2DM and PTB compared to those in other groups. IL-10 can be secreted by a variety of cells in the body, and can effectively regulate the function of monocytes/macrophages, as a negative regulator of cell-mediated immune response, and can inhibit the production of pro-inflammatory factors by monocytes and macrophages. IL-10 has a two-way immunomodulatory effect, that can be suppressed by antigen-presenting cells (APCs) and is negatively regulated by T cells. It negatively regulates immune responses in the tumor environment. In addition, IL-10 can stimulate T and B lymphocytes, and IL-10 can also stimulate T and B lymphocytes in patients with tumor. Some studies have shown that an increase in cellular IL-10 secretion is related to the susceptibility to PTB (29). The serum level of IL-10 in patients with PTB is higher than that in healthy controls, which inhibits the production of pro-inflammatory factors by monocytes and macrophages, weakens the antigen presentation of macrophages, and reduces the clearance of M. tuberculosis by the immune system. IL-10 is associated with an inflammatory response and insulin resistance in T2DM (30), but it is not clear whether higher IL-10 levels inhibit the occurrence of T2DM by reducing the production of pro-inflammatory cytokines, or whether increased IL-10 levels in patients with T2DM lead to a compensatory response to increased pro-inflammatory mediators (mainly tumor necrosis factor-α and IL-6) (31).

The proliferation and differentiation of immune cells can be promoted by IL-6, thereby improving their ability to secrete antibodies and enhance immune activity. Meanwhile, IL-6 can promote the release of relevant provocative mediators participating in immunopathological processes (28). However, IL-6 mainly plays a role in the early stages of the inflammatory reaction (32). Likewise, the serum IL-6 levels in the T2DM plus PTB group were significantly higher than those in the T2DM group, indicating the presence of a PTB-reinforced inflammatory response in these patients.

The limitations of this study are as follows: First, although the study participants were age- and sex-matched, the sample size of each group was relatively small. Second, it was not feasible to isolate and culture T cells from patients with PTB to evaluate the effect of IL-10 on T cell expansion and activation. Third, neither fasting insulin nor C-peptide levels were determined in patients with T2DM combined with PTB.

In conclusion, the presence of a PTB infection maintained greater hyperglycaemia and monocyte numbers in patients with T2DM than in with only T2DM. Treatment of T2DM becomes more complicated and challenging when patients are infected with PTB. Better precision medicine-based therapies are required to control hyperglycaemia and inflammation in patients with T2DM combined with PTB.

## Data availability statement

The original contributions presented in the study are included in the article/supplementary materials. Further inquiries can be directed to the corresponding authors.

## Ethics statement

The studies involving human participants were reviewed and approved by Institutional Review Board of the Heilongjiang Provincial Hospital. The patients/participants provided their written informed consent to participate in this study.

## Author contributions

HC, LS, and YL contributed to conception and design of the study. KZ and EM organized the database. JB performed the statistical analysis. HC and SL wrote the first draft of the manuscript. JB, KZ, YL, and EM wrote sections of the manuscript. All authors contributed to the article and approved the submitted version.

## Funding

This study was supported by Natural Science Foundation of Heilongjiang Province of China (program number: LH2021H069), Research Project of Heilongjiang Provincial Health Commission of China (program number: 20211212020239), and the Heilongjiang Postdoctoral Fund (NO.LBH-Z16235).

## Acknowledgments

The authors thank the Heilongjiang Postdoctoral Fund (NO.LBH-Z16235) for providing financial support. The authors thank Editage (www.editage.com) for English language editing.

## Conflict of interest

The authors declare that the research was conducted in the absence of any commercial or financial relationships that could be construed as a potential conflict of interest.

## Publisher's note

All claims expressed in this article are solely those of the authors and do not necessarily represent those of their affiliated organizations, or those of the publisher, the editors and the reviewers. Any product that may be evaluated in this article, or claim that may be made by its manufacturer, is not guaranteed or endorsed by the publisher.
